# Primary Abdominal Ectopic Pregnancy Involving the Spleen: A Case Study

**DOI:** 10.1155/crog/5344541

**Published:** 2026-07-14

**Authors:** Massimiliano Fambrini, Elisa Farsi, Eleonora Nardi, Elena Nocentini, Flavia Sorbi, Francesca Castiglione

**Affiliations:** ^1^ Department of Experimental, Clinical and Biomedical Sciences “Mario Serio”, University of Florence, Florence, Italy, unifi.it; ^2^ Department of Health Sciences, Section of Pathology, University of Florence, Florence, Italy, unifi.it

**Keywords:** abdominal, accessory spleen, case report, ectopic pregnancy, histologic examination

## Abstract

**Introduction:**

Ectopic pregnancy, affecting 1%–2% of pregnancies, is a major cause of first‐trimester maternal morbidity and mortality, most commonly occurring in the fallopian tubes. Abdominal ectopic pregnancy is rare (< 1%) but highly dangerous, with risk of hemorrhage, infection, and misdiagnosis. Splenic implantation is exceptionally rare. Diagnosis relies on ultrasound, MRI, and beta‐hCG levels, though symptoms are often nonspecific. Risk factors include tubal disease, infections, prior surgery, and assisted reproduction. Early diagnosis is critical, as maternal and perinatal mortality are high. Management is usually surgical, with laparoscopy preferred in stable early cases and laparotomy in more advanced or complicated presentations.

**Case Presentation:**

A 29‐year‐old gravida 2 para 1 at 5 + 4 weeks presented with mild vaginal bleeding and stable vital signs. Serum beta‐hCG levels showed an abnormal rise followed by a plateau. Transvaginal ultrasound revealed no intrauterine pregnancy, suggesting a pregnancy of unknown location, with a suspicious right‐sided adnexal finding. A second‐level ultrasound identified a vascularized multilocular mass near the anterior uterine recess, raising concern for ectopic or abdominal pregnancy. Due to diagnostic uncertainty and plateauing beta‐hCG, exploratory laparoscopy was performed. The pelvis appeared normal, but two vascularized formations were found on the prevesical peritoneum and excised. Postoperatively, beta‐hCG levels declined significantly. Histological analysis confirmed first‐trimester chorionic villi and trophoblast cells within splenic tissue, consistent with ectopic pregnancy implanted in splenosis. The patient recovered well and was discharged in stable condition with near‐complete resolution of beta‐hCG levels on follow‐up.

**Discussion:**

Abdominal pregnancy is a rare, life‐threatening ectopic pregnancy with a challenging diagnosis. In this case, inconclusive imaging and plateauing beta‐hCG prompted laparoscopy, confirming prevesical implantation. Prior splenectomy may have contributed. Surgical removal led to resolution, highlighting the importance of systematic evaluation, clinical suspicion, and timely intervention in atypical ectopic pregnancies.

## 1. Introduction

Ectopic pregnancy (EP) is one of the most important causes of morbidity and maternal mortality in the first trimester [1–3]. It is defined as the implantation and growth of a blastocyst outside the endometrium [4, 5], and it accounts for 1%–2% of all pregnancies. Among them, in more than 90% of cases the implantation site is in the fallopian tube [6].

Instead, abdominal ectopic pregnancy (AEP) is a rare form (< 1%) of EP occurring when the fertilized egg implants in the peritoneal cavity, including the omentum, and abdominal organs such as the spleen and intestine [2, 7]. In developed countries, abdominal pregnancy is extremely rare [8]; whereas, in developing countries, it is more common occurring in advanced stages due to lack of medical care, poor antenatal healthcare provision, and low socioeconomic patient status [9, 10].

Reports of splenic ectopic pregnancy (SEP) are rare, representing a high‐mortality condition [11].

In 1942, Studdiford established three criteria for diagnosis of a primary peritoneal pregnancy: the presence of normal tubes and ovaries, no evidence of utero‐peritoneal fistula, and the presence of a pregnancy related exclusively to the peritoneal surface and early enough in gestation to eliminate the possibility of secondary implantation after primary nidation of the tube [12].

Instead, a classification relevant from treatment classifies peritoneal EP in early (less than 20 weeks of gestation) or advanced (more than 20 weeks of gestation) [13].

Abdominal pregnancy can also be classified into primary and secondary [14].

AEP has a huge potential for maternal mortality and morbidity [7] [15].

The high maternal mortality rate is especially concerning since abdominal EPs are commonly misdiagnosed [16] and they have an intrinsic increased risk of fatal intraperitoneal hemorrhage [17].

The risk of hemorrhage is mainly due to the fact that the placenta frequently implants on highly vascular structures in the abdominal cavity.

Maternal morbidity can also be substantial, with high incidences of pelvic abscess, peritonitis, and sepsis caused by retained placental remnants [15], whereas the morbidity associated with AEP includes infection and infertility [17].

Abdominal pregnancies may mimic other conditions, such as acute appendicitis, that can delay the diagnosis. In addition, if it occurs in the pelvic cavity, it can be misdiagnosed with tubal EP [6].

The symptoms are different, nonspecific and vary according to the site of implantation; abdominal pain, vaginal bleeding and nausea, vomiting, and general malaise are common [8].

The exact cause of an abdominal EP is not clear, but risk factors that may contribute include a pathology of the fallopian tubes, pelvic infections, a previous abdominal surgery, and the use of fertility treatments.

Different cases of abdominal EPs reaching term have been reported in the literature; nevertheless, termination of pregnancy is recommended when the diagnosis is made before 24 weeks due to the high mortality rates associated with massive hemorrhage [18].

Thus, the rate of perinatal mortality is also very high, ranging from 40% to 95% [19]. Generally, radiologic methods that aid in diagnosis include pelvic ultrasound and magnetic resonance imaging (MRI).

Key ultrasonographic indicators include the absence of the myometrial wall between the gestational sac and the bladder, an unusual fetal position with parts of the fetus abnormally close to the abdominal wall, and occasionally abnormal placental vascularization [7]. MRI, on the other hand, can provide detailed visualization, helping to assess the relationship of the placenta to adjacent organs and, in some cases, identify placental invasion [20].

In addition, beta − human chorionic gonadotropin levels (beta − hCG) > 1500 mIU/mL, in the early stage of pregnancy, with an ultrasound finding of absence of intrauterine gestational sac should arouse suspicion of EPs [17].

Surgical intervention is the typical treatment in cases of EP, even if it can be very challenging depending on the location of the implantation site.

Abdominal pregnancies before 24 weeks of gestation are generally treated with laparotomy [17]. The laparoscopic approach is generally preferred when the patient is hemodynamically stable, shows no signs of shock, the pregnancy is at an early stage, and the implantation site is easily accessible [4].

## 2. Case Presentation

On October 2024 a 29‐year‐old, gravida 2 para 1 at 5 weeks + 4 days gestational age (calculated from the last menstrual period), presented to the Obstetric and Gynecological Emergency Department of Careggi University Hospital, Florence, due to minimal vaginal bleeding that started the previous day. The bleeding was not associated with pelvic pain and was unrelated to recent sexual activity or gynecological vaginal examinations.

The pregnancy was spontaneous. Her obstetric history accounted for one spontaneous vaginal delivery in 2022 at term, uncomplicated pregnancy.

The patient′s medical history included a low body weight (BMI: 17.9), a negative cervical cancer screening, and a posttraumatic total splenectomy in 2015.

She was not on any chronic medication.

**Figure 1:**
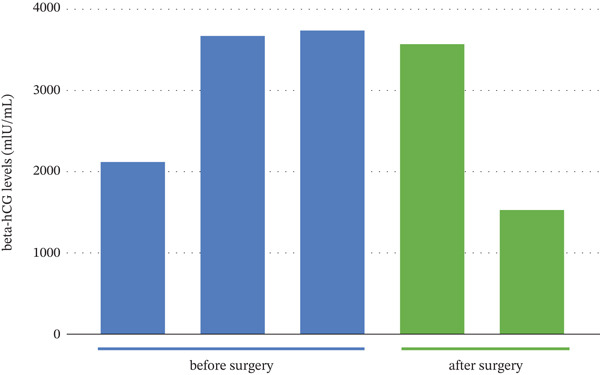
Beta‐hCG levels before and after surgery.

The results of recent serum beta‐hCG levels, as requested by her primary gynecologist in prior weeks, showed a gradual increase from 2111 to 3658 mIU/mL over time (Figure 1).

On initial examination, the patient was alert and in good clinical condition, and vital signs were stable. Physical examination revealed a soft abdomen without signs of peritoneal irritation. During vaginal examination, the cervix was posterior, of firm consistency, uneffaced and closed, whereas on speculum examination, the cervix and vaginal walls appeared macroscopically normal, with traces of blood in the vagina.

On transvaginal office‐based ultrasound, the uterus was retroverted, with a globular morphology and a heterogeneous myometrial structure suggestive of adenomyosis. The endometrial lining was homogeneous. No intrauterine gestational sac was visualized. The right ovary appeared normal with microfollicular features, and an adjacent dilated ipsilateral tube was noted, but no trophoblastic tissue was visible. The left adnexa appeared regular. There was a minimal amount of fluid in the pouch of Douglas, and mild tenderness was noted upon probe pressure in the right adnexal region.

The patient was admitted for further evaluation due to a suspected pregnancy of unknown location (PUL).

Monitoring of vital signs, assessment of vaginal bleeding, and a repeat serum beta‐hCG measurement were performed. The following day, the patient was kept under observation.

**Figure 2:**
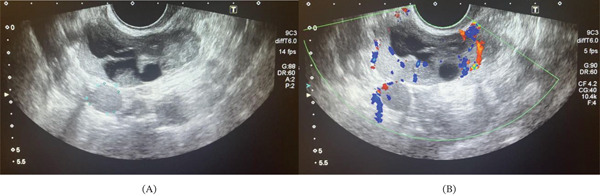
Ultrasound appearance of neoformation located in prevescical peritoneum (abdominal pregnancy) on (A) greyscale and (B) power Doppler imaging.

A second‐level pelvic ultrasound was performed, which showed an elongated image in the anterior uterine recess on the right side, multilocular with a peripheral and central solid component, richly vascularized on color Doppler CS3, measuring 51 × 20 mm. Medially to this, but apparently not communicating with it, a unilocular cyst measuring 20 × 15 mm was noted with sparse peripheral vascularization (CS2). (Figure 2).

These findings were of unclear diagnostic interpretation (right tubal pregnancy vs. abdominal pregnancy).

The vaginal bleeding remained stable, as did her clinical condition. The two subsequent beta‐hCG measurements, taken at admission and the following day, showed a plateau trend: 16/10: 3,730.0 U/L and 17/10: 3,563.0 U/L. The decision to perform an exploratory laparoscopy was taken.

**Figure 3:**
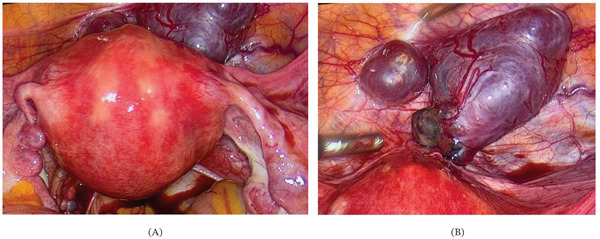
(A–B) Representative images taken during the exploratory laparoscopy. (A) Uterus and adnexa were macroscopically normal. (B) The two neoformations on the prevescical peritoneum.

Intraoperatively, the surgeon described the presence of diffuse omento‐parietal adhesions, which significantly hindered the exploration of the upper abdomen. The pelvis was free from these adhesions, allowing visualization of the uterus and adnexa, which appeared macroscopically normal. Two subcentimeter implants, suspicious for splenosis or accessory spleens, were observed in the omentum and prevesical peritoneum. Given the patient′s history of traumatic splenectomy, these were clinically suggestive of splenosis. They were excised and sent for histological examination. At the prevesical peritoneum, in the area of the vesicouterine fold, two adjacent and adherent formations were identified, but not apparently communicating. One was round with smooth walls, approximately 2 cm in diameter, and the other was multilobulated, with a maximum diameter of about 4 cm, richly vascularized, containing both blood and solid material resembling trophoblastic tissue, seemingly consistent with EP localization. The entire neoformation was adherent to the prevesical peritoneum, from which it was cautiously dissected (Figure 3).

A significant decrease in beta‐hCG was observed 12 h after the surgery, with a value of 1535,0 U/L. The patient recovered well and was discharged the day after the surgery in good general conditions, with another planned repetition of serum beta‐hCG dosage to do in 10 days that was almost negative.

On gross examination, the neoformation appeared as multiple blood fragments in the complex of 50 × 40 × 15 mm. The fragments were submitted for histological evaluation and, after formalin‐fixation, they were stained with hematoxylin and eosin (H&E).

**Figure 4:**
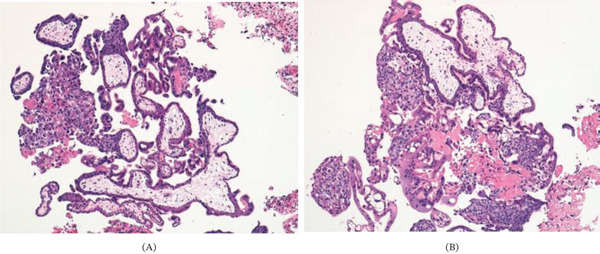
(A–B) Representative histologic images showing large chorionic villi covered by two layers of cells (cytotrophoblast and syncytiotrophoblast) and no prominent blood vessels within (first‐trimester morphology) admixed with trophoblast cells ((A–B) H&E magnification 10×).

**Figure 5:**
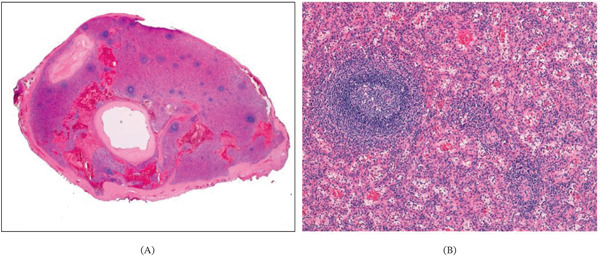
(A–B) Representative histologic sections showing normal appearance of the spleen (white and red pulp) ((A) H&E magnification 2×; (B) H&E magnification 10×).

Histologically, chorionic villi with a first‐trimester morphology and trophoblast cells were identified in a context of chronic inflammation with abundant blood clots; in addition, lymphoid follicles and red pulp forming the majority of splenic parenchyma were identified (Figures 4 and 5).

## 3. Discussion

This case highlights the diagnostic and management challenges of an AEP in an unusual location, presenting as a PUL. Abdominal pregnancy is a rare and life‐threatening form of EP, with an estimated incidence of 1 per 10,000 births [21], being responsible for about 4% of all pregnancy‐related mortalities [22]. In our patient, clinical and radiologic findings were initially inconclusive, emphasizing the importance of systematic evaluation, including repeat beta‐hCG measurements, advanced imaging techniques, and, when necessary, surgical exploration.

The patient′s initial presentation, characterized by minimal vaginal bleeding without pelvic pain or hemodynamic instability, is a common but nonspecific feature of EP. The absence of an intrauterine gestational sac on transvaginal ultrasound raised suspicion of an EP, whereas the plateauing beta‐hCG levels suggested a nonviable pregnancy. However, the unusual findings on second‐level ultrasound, such as a richly vascularized multilocular structure in the anterior uterine recess, complicated the interpretation, prompting the consideration of differential diagnoses, including tubal and abdominal pregnancies.

MRI has been described as a useful tool in evaluating EPs, particularly in complex cases where precise localization is needed. However, in this case, exploratory laparoscopy was prioritized due to persistent diagnostic uncertainty and the risk of intra‐abdominal bleeding.

Laparoscopy confirmed an abdominal EP located in the prevesical peritoneum, an exceedingly rare site. The significant omento‐parietal adhesions observed intraoperatively likely resulted from the patient’s prior splenectomy, which may have contributed to the ectopic implantation by altering normal peritoneal anatomy. The histological findings of chorionic villi and trophoblastic tissue in the excised mass confirmed the diagnosis.

The presence of splenic tissue in the peritoneal cavity in this case is particularly noteworthy. Although congenital accessory spleens occur in approximately 10%–15% of the population, splenosis, the autotransplantation of splenic pulp following splenic rupture or surgery, is a distinct possibility here. Splenosis typically results in multiple sessile implants that lack a formal capsule and receive blood supply from adjacent tissues. In this patient, the previous traumatic splenectomy likely led to the seeding of splenic tissue throughout the peritoneum. Interestingly, the EP implanted directly onto or adjacent to these splenic foci, suggesting that the highly vascular nature of splenosis may have provided an inviting environment for trophoblastic nidation.

The decision to proceed with surgery was guided by the plateauing beta‐hCG levels, unclear imaging findings, and the potential risks of continued expectant management. Laparoscopy is considered the gold standard for diagnosing and managing EPs, particularly in ambiguous cases like this one. The successful removal of the EP and subsequent decline in beta‐hCG confirmed complete resolution. The patient′s uneventful recovery underscores the value of timely intervention.

This case underscores the need for a high index of suspicion and a multidisciplinary approach in managing atypical presentations of EP. Key points include the following:

• Importance of imaging: Although ultrasonography is the first‐line diagnostic tool, complex cases may require advanced imaging or surgical exploration for definitive diagnosis.
• Role of prior surgery: The patient′s history of splenectomy likely contributed to the ectopic implantation, highlighting the need to consider prior surgical interventions when evaluating atypical EPs.
• Surgical expertise: The ability to identify and safely excise EP in rare and challenging locations demonstrates the critical role of experienced surgical teams in managing such cases.

## 4. Conclusion

Abdominal EPs, especially in rare locations such as the prevesical peritoneum, remain diagnostic and therapeutic challenges. This case emphasizes the value of systematic evaluation, timely intervention, and a multidisciplinary approach in achieving favorable outcomes. Future research should explore the relationship between prior surgeries and ectopic implantation to better understand the mechanisms and guide preventive strategies.

**Nomenclature**
EP: ectopic pregnancy
AEP: abdominal ectopic pregnancy
SEP: splenic ectopic pregnancy
BMI: body mass index
*β*‐HCG: beta‐human chorionic gonadotropin
PUL: pregnancy of unknown location
H&E: hematoxylin and eosin

## Author Contributions

Conceptualization: Elisa Farsi and Eleonora Nardi; data acquisition: Elisa Farsi, Eleonora Nardi, and Elena Nocentini; writing: Elisa Farsi and Eleonora Nardi; review: Massimiliano Fambrini, Francesca Castiglione, and Flavia Sorbi; validation: Massimiliano Fambrini, Elisa Farsi, Eleonora Nardi, Elena Nocentini, Flavia Sorbi, and Francesca Castiglione.

## Funding

No funding was received for this manuscript. Open access publishing facilitated by Universita degli Studi di Firenze, as part of the Wiley ‐ CRUI‐CARE agreement.

## Disclosure

All authors have read and approved the final version of the manuscript. Elisa Farsi affirms that this manuscript is an honest, accurate, and transparent account of the study being reported; that no important aspects of the study have been omitted; and that any discrepancies from the study have been explained.

## Ethics Statement

The present study complied with the ethical principles for medical research involving human subjects, as outlined in the World Medical Association Declaration of Helsinki. Written informed consent was obtained from the patient for publication of this case report and any accompanying images. All clinical, instrumental, and histopathological data were fully anonymized prior to publication to ensure patient confidentiality.

## Conflicts of Interest

The authors declare no conflicts of interest.

## Supporting information


**Supporting Information** Additional supporting information can be found online in the Supporting Information section. The CARE reporting checklist is available as Supporting Information.

## Data Availability

The authors confirm that the data supporting the findings of this study are available within the article.
