# Supplementary material for: Primary Abdominal Ectopic Pregnancy Involving the Spleen: A Case Study
Source: Case Rep Obstet Gynecol. 2026 Jul 14;2026:5344541. doi: 10.1155/crog/5344541 (PMC13369295; doi:10.1155/crog/5344541)
Supplement: Supplementary file 1 — Supporting Information Additional supporting information can be found online in the Supporting Information section. The CARE reporting checklist is available as Supporting Information. [file CROG-2026-5344541-s001.pdf]

CARE case report guidelines

CARE Checklist of information to include when writing a case report

(cc) BY-NC-ND

Topic	Item	Checklist item description	Reported on Line
Title	1	The diagnosis or intervention of primary focus followed by the words "case report"	1
Key Words	2	2 to 5 key words that identify diagnoses or interventions in this case report, including "case report"	66-67
Abstract (no references)	3a	Introduction: What is unique about this case and what does it add to the scientific literature?	36-38
	3b	Main symptoms and/or important clinical findings	43-44
	3c	The main diagnoses, therapeutic interventions, and outcomes	45-49
	3d	Conclusion—What is the main "take-away" lesson(s) from this case?	57-59
Introduction	4	One or two paragraphs summarizing why this case is unique (**may include references**)	105; 111
Patient Information	5a	De-identified patient specific information.	157
	5b	Primary concerns and symptoms of the patient.	159-160
	5c	Medical, family, and psycho-social history including relevant genetic information	164-168
	5d	Relevant past interventions with outcomes	165
Clinical Findings	6	Describe significant physical examination (PE) and important clinical findings.	170-176
Timeline	7	Historical and current information from this episode of care organized as a timeline	159-160
Diagnostic Assessment	8a	Diagnostic testing (such as PE, laboratory testing, imaging, surveys).	169-171
	8b	Diagnostic challenges (such as access to testing, financial, or cultural)	N/A
	8c	Diagnosis (including other diagnoses considered)	192-193
	8d	Prognosis (such as staging in oncology) where applicable	194
Therapeutic Intervention	9a	Types of therapeutic intervention (such as pharmacologic, surgical, preventive, self-care)	196
	9b	Administration of therapeutic intervention (such as dosage, strength, duration)	196
	9c	Changes in therapeutic intervention (with rationale)	N/A
Follow-up and Outcomes	10a	Clinician and patient-assessed outcomes (if available)	211-213
	10b	Important follow-up diagnostic and other test results	210-213
	10c	Intervention adherence and tolerability (How was this assessed?)	N/A
	10d	Adverse and unanticipated events	N/A
Discussion	11a	A scientific discussion of the strengths AND limitations associated with this case report	223-224
	11b	Discussion of the relevant medical literature **with references**.	265-281
	11c	The scientific rationale for any conclusions (including assessment of possible causes)	274-281
	11d	The primary "take-away" lessons of this case report (without references) in a one paragraph conclusion	284-285
Patient Perspective	12	The patient should share their perspective in one to two paragraphs on the treatment(s) they received	N/A
Informed Consent	13	Did the patient give informed consent? Please provide if requested	Yes ☒ No ☐
